# Competition quenching strategies reduce antibiotic tolerance in polymicrobial biofilms

**DOI:** 10.1038/s41522-024-00489-6

**Published:** 2024-03-19

**Authors:** Bram Lories, Tom E. R. Belpaire, Bart Smeets, Hans P. Steenackers

**Affiliations:** 1 Department of Microbial and Molecular Systems, Centre of Microbial and Plant Genetics (CMPG), KU Leuven Leuven, Belgium; 2 Division of Mechatronics, Biostatistics, and Sensors (MeBioS), Department of Biosystems, KU Leuven Leuven, Belgium

**Keywords:** Antimicrobials, Microbial ecology, Biofilms, Pathogens

## Abstract

Bacteria typically live in dense communities where they are surrounded by other species and compete for a limited amount of resources. These competitive interactions can induce defensive responses that also protect against antimicrobials, potentially complicating the antimicrobial treatment of pathogens residing in polymicrobial consortia. Therefore, we evaluate the potential of alternative antivirulence strategies that quench this response to competition. We test three competition quenching approaches: (i) interference with the attack mechanism of surrounding competitors, (ii) inhibition of the stress response systems that detect competition, and (iii) reduction of the overall level of competition in the community by lowering the population density. We show that either strategy can prevent the induction of antimicrobial tolerance of *Salmonella* Typhimurium in response to competitors. Competition quenching strategies can thus reduce tolerance of pathogens residing in polymicrobial communities and could contribute to the improved eradication of these pathogens via traditional methods.

## Introduction

Bacteria often live embedded in diverse and complex communities. The social interactions in these polymicrobial consortia can result in enhanced levels of virulence and antimicrobial tolerance, complicating the treatment of infections and the eradication of undesirable bacteria in industrial settings^1–3^. An increase in tolerance in mixed culture communities has been reported for several common pathogens, including *Salmonella*, *Listeria monocytogenes*, *Staphylococcus aureus, Pseudomonas aeruginosa,* and uropathogenic *Escherichia coli*^4–7^. In some cases, interspecies interactions can even increase the number of cells surviving the antimicrobial treatment by a 100-fold^4^.

Cooperation is common between highly related bacteria and interspecies cooperation can occur by, for example, detoxifying harmful environments^8^ or exchanging metabolic byproducts^9^. However, in most cases, these polymicrobial consortia are predominantly shaped by competitive interactions^10–12^ as the ecological conditions that favor helping other strains are highly restrictive^13,14^. Consequently, competition often underlies the increased virulence and tolerance of polymicrobial communities^5,12,15–17^. Due to the prevalence of competition, it has been suggested that bacteria have adapted their stress response systems to detect the damage caused by competition and respond accordingly. This idea is central to the recent ‘competition sensing’ hypothesis, which posits that stress responses play an important role for microbes to detect their social environment^18^. Activation of stress response systems in response to competition has indeed been observed in several species and against a variety of attack mechanisms^19–22^. In addition, bacteria across the phylogenetic tree regulate the production of bacteriocins and antibiotics by stress responses that sense nutrient limitation and cell damage, both stressors commonly associated with bacterial competition^18,23^. This regulation of bacterial weapons by stress response systems further supports that stress responses play an important role in competition. However, these stress response systems do not only regulate competitive behavior, they also coordinate various tolerance and virulence phenotypes^21,24,25^. Activation of stress response systems by competition could thus pleiotropically activate these phenotypes, which could -at least partly- underlie the enhanced tolerance and virulence in polymicrobial communties.

In support of this hypothesis, we previously showed that several of the stress response systems of *Salmonella* Typhimurium are activated by the Type VI secretion system (T6SS) of surrounding competitors. Activation of these stress responses in co-culture conditions led to enhanced invasion in gut epithelial cells, increased biofilm matrix production and increased efflux of antibiotics^5^. This response is expected to aid in the competition with other strains since increased biofilm formation and efflux can protect against antimicrobials produced by surrounding competitors, while it has been suggested that epithelial invasion can trigger an inflammatory response and create favorable conditions for the *Salmonella* cells that remain in the gut lumen to compete with the microbiome^5^. However, next to the potential role in competition, these same phenotypes increase the antimicrobial tolerance and virulence^26–28^, highlighting that not only pleiotropic effects of stress response activation but also the defensive responses against competition themselves could contribute to the severity of polymicrobial infections. Therefore, we here propose an alternative antimicrobial strategy that interferes with competition in order to weaken the virulence and tolerance of pathogens residing in mixed culture communities and potentially improve the clinical outcome of polymicrobial infections^29–31^.

**Fig. 1:**
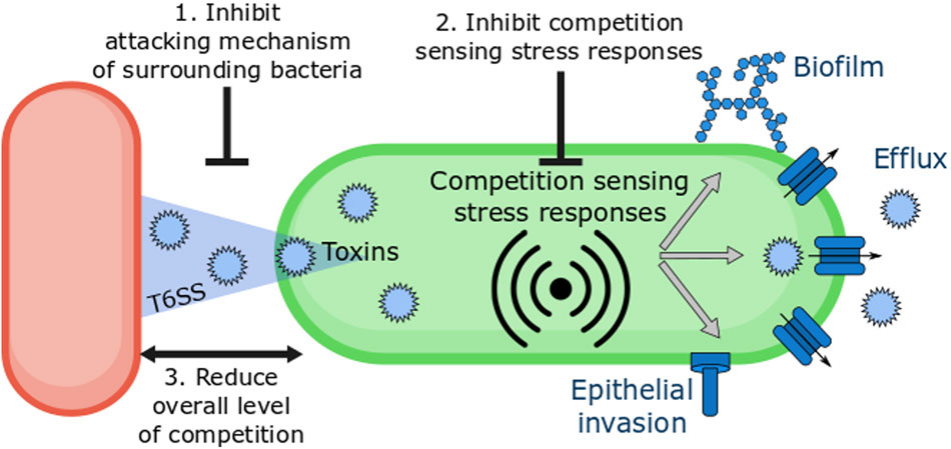
Schematic overview of the explored competition quenching strategies. The defensive response to competition can be targeted by either (1) inhibiting the attack mechanism of surrounding competitors, (2) interfering with the stress response systems that detect competition, or (3) reducing the overall level of competition in the community by decreasing the density.

The proposed strategy of targeting inter-strain competition shows several analogies with quorum quenching therapies that interfere with the quorum sensing systems regulating cooperation between bacteria of the same strain^32,33^. Therefore, we call this strategy ‘competition quenching’. The potency of each approach would be determined by the relative contribution of inter-strain competition and intra-strain cooperation to the pathogenicity and tolerance of a pathogen in a certain community. Similar to quorum quenching inhibiting either the signal supply or the response to the signal^33,34^, competition quenching strategies can also be designed to interfere with the competitive cascade at different levels. One can either (i) interfere with the attack mechanism of surrounding competitors, (ii) inhibit the stress response systems that detect competition directly^18^, or (iii) reduce the overall level of competition in the community (Fig. 1).

We here explore the potential of these different competition quenching strategies. We show that either approach can abolish the enhanced antimicrobial tolerance of *Salmonella* residing in a competitive consortium. In addition, we show that decreasing the level of competition can also reduce the expression of pathways regulating other virulence-associated traits such as epithelial invasion and biofilm formation. Our findings thus support that competition quenching strategies can facilitate the eradication of pathogens living in polymicrobial communities.

## Results

### Genetically inhibiting the T6SS activity in surrounding competitors abolishes the increased antimicrobial tolerance in polymicrobial biofilms

**Fig. 2:**
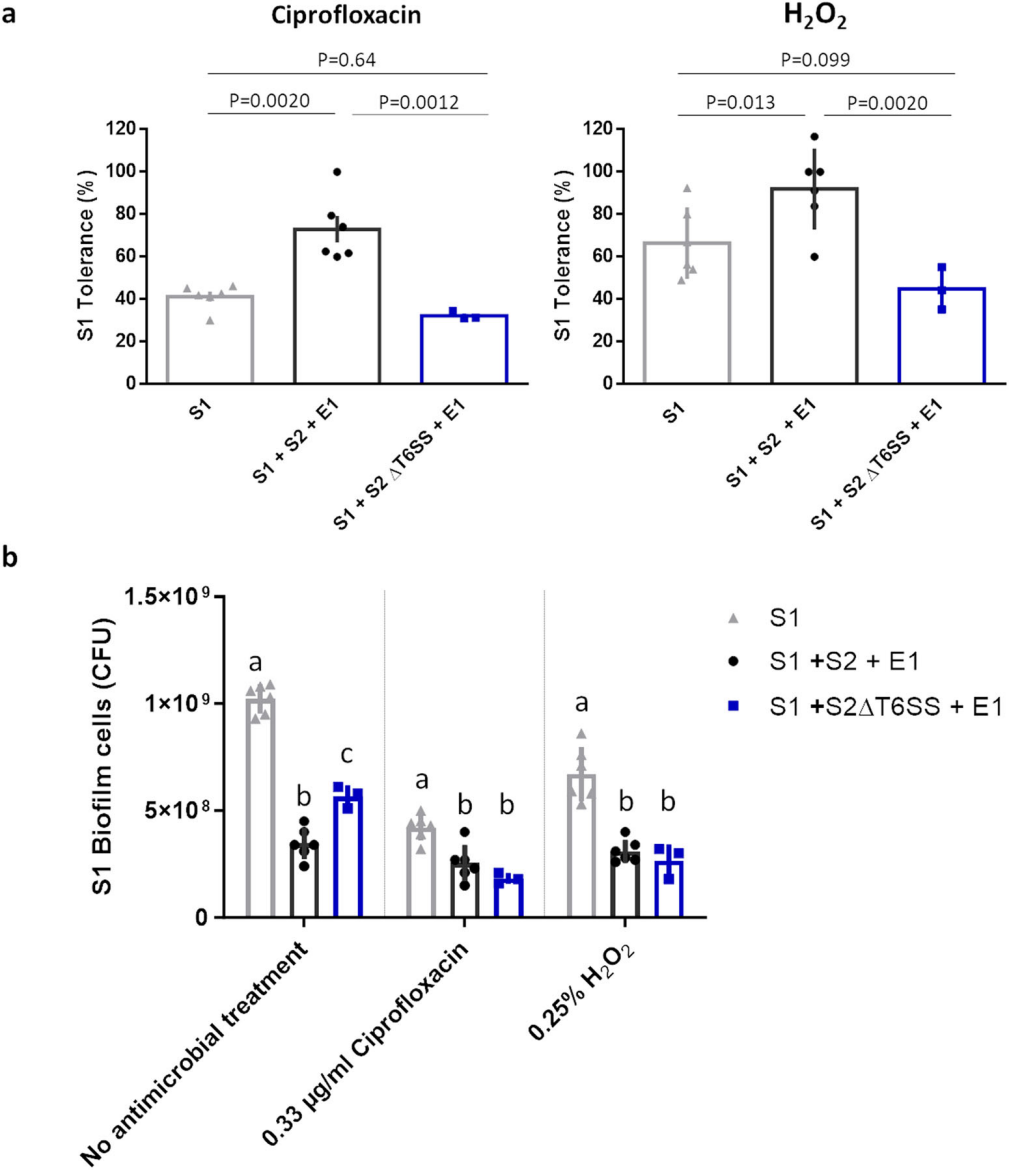
Inactivation of T6SS prevents the induction of antimicrobial tolerance in polymicrobial biofilms, but results in a similar number of S1 cells surviving the antimicrobial treatments due to the lowered competition. **a** The tolerance of S1 towards common antimicrobials is no longer enhanced in mixed-culture conditions if the T6SS of S2 is inactivated. S1 tolerance is calculated as the ratio between the number of antimicrobial treated and mock treated S1 biofilm cells, either in monoculture or mixed culture conditions. The mean and standard deviation of three to six biological repeats are shown. *P* values were calculated via a two-way ANOVA with Tukey multiple comparisons corrections. **b** A similar level of S1 cells survive the antimicrobial treatments in both mixed-culture communities. The mean and standard deviation of three to six biological repeats are shown. Significant differences are calculated via a two-way ANOVA with Tukey multiple comparisons corrections. Different superscript letters indicate significant number of S1 cells surviving treatment, determined per antimicrobial treatment (*P* < 0.05).

We previously showed in a model community consisting of two *Salmonella* (_S1: *S*. Typhimurium SL1344, S2: *S*. Typhimurium ATCC14028_) strains and one *E. coli* (_E1: *E. coli* MG1655_) strain that a T6SS-mediated attack by S2 activates the stress response systems of S1^5^. In response to this competition, the S1 strain showed increased antibiotic tolerance, biofilm formation and epithelial invasion. In addition, the S2 ΔT6SS deletion mutant did not activate the S1 stress responses and the downstream pathways involved in antibiotic tolerance, biofilm formation and epithelial invasion were no longer upregulated^5^. However, we did not study how inactivation of the T6SS influenced the response to competition at the phenotypic level. Therefore, we first determined the validity of the competition quenching concept by measuring the effect of knocking out the T6SS in S2 on the antimicrobial tolerance of S1. We opted to focus on the tolerance phenotype instead of biofilm formation or invasion since tolerance can be measured unambiguously in mixed culture conditions. In contrast, biofilm assays do not allow to differentiate between the biofilm matrix formed by the different strains as they produce highly similar matrix components^35,36^, while the epithelial cell line model systems required to study invasion in vitro do not support prolonged exposure to pathogens such as *Salmonella*^37–39^. To quantify the induction of antimicrobial tolerance in polymicrobial conditions, we treated mature monoculture and mixed culture biofilms with hydrogen peroxide or ciprofloxacin (Fig. 2a, Supplementary Fig. 1). These two antimicrobials are commonly used to combat *Salmonella* in respectively industrial^40^ or clinical^41^ settings. In line with our previous results, S1 showed a higher tolerance towards both antimicrobials in the presence of competitors^5^. The tolerance towards ciprofloxacin was 1.75 times greater in mixed culture biofilms, while the tolerance to hydrogen peroxide was 1.5 times higher. However, in absence of an active T6SS in S2, the enhanced tolerance of S1 in polymicrobial conditions was found to be completely abolished, further supporting that a defensive response against T6SS is the lone driver of the enhanced tolerance in our model community^5^. Moreover, these findings confirm that interfering with competition can reduce the tolerance of pathogens in polymicrobial communities. However, the lack of an active T6SS in S2 increased the number of S1 cells in the untreated mixed culture biofilm. Despite the reduced tolerance of S1 in the community where the T6SS of S2 is inactivated, this reduction in competitive inhibition resulted in a similar absolute number of S1 cells surviving the antimicrobial treatments as in the wild type community (Fig. 2b, Supplementary Fig. 1). Therefore, we did not test chemical inhibitors of the T6SS but instead explored a strategy that directly inhibits the competition sensing systems and does not allow the pathogen to thrive initially.

### Direct RpoS inhibition interferes with the enhanced tolerance in response to competition

**Fig. 3:**
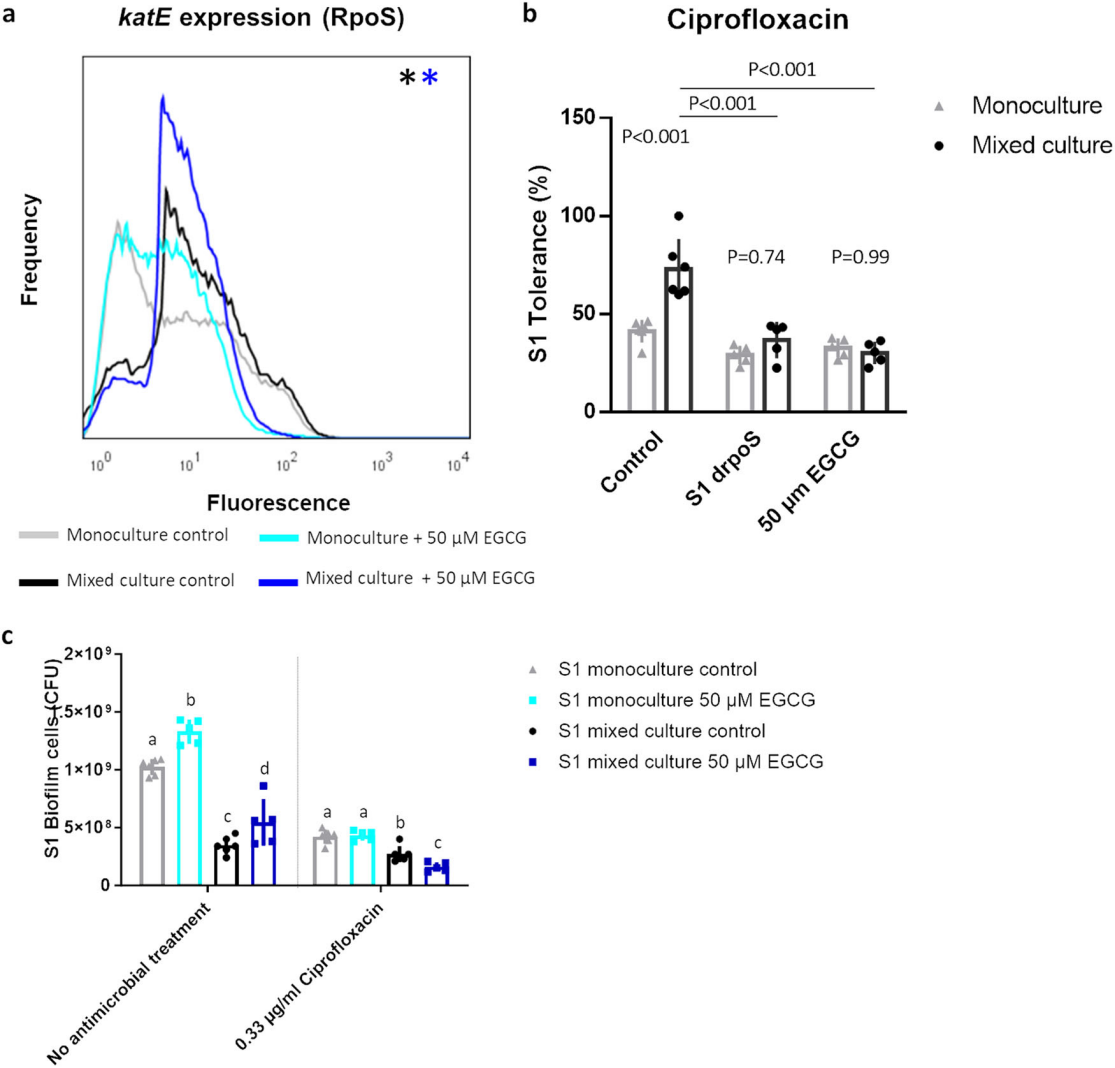
The RpoS inhibitor EGCG abolishes the increased tolerance against ciprofloxacin in mixed-culture conditions. **a** 50 µM ECGC reduces the expression of the *katE* reporter gene for RpoS activity, both in monoculture and mixed culture conditions. The FACS profiles show the population distribution of S1 fluorescence under the different conditions. In each condition, 100 000 cells are analyzed. Significant differences between expression profiles are determined via probability binning as described in materials & methods. Significant differences between monoculture and mixed culture conditions with the addition of DMSO are indicated with a black asterisk whereas differences between monoculture and mixed culture conditions in the presence of EGCG are indicated with an blue asterisk. One representative repeat of two biological repeats is shown. **b** Both the genomic deletion of *rpoS* and the inhibition of RpoS activity via addition of 50 µM EGCG interfere with the increased ciprofloxacin tolerance of S1 in mixed culture conditions. The mean and standard deviation of five to six biological repeats are shown. *P* values were calculated via a two-way ANOVA with Tukey multiple comparisons corrections. **c** A combination treatment of EGCG and ciprofloxacin results in the lowest absolute number of S1 cells in mixed culture conditions. The mean and standard deviation of three to six biological repeats are shown. Significant differences are calculated via a two-way ANOVA with Tukey multiple comparisons corrections. Different superscript letters indicate significant number of S1 cells surviving treatment, determined per antimicrobial treatment (*P* < 0.05).

We previously observed that the strong competition experienced by S1 in the polymicrobial biofilm activated the general stress response mediated by RpoS, the two-component system PhoPQ, and the oxidative stress response system SoxRS. Activation of these stress response systems resulted in an increased expression of downstream genes regulating biofilm formation, efflux and epithelial invasion. Importantly, genomic deletion of either the RpoS or SoxRS stress response prevented the induction of all three downstream pathways^5^. Therefore, we studied whether chemically inhibiting a single competition sensing stress response system could also reduce the tolerance of S1 in mixed culture conditions. We focused on the general stress response since a compound that inhibits this stress response system has been identified previously. Epigallocatechin gallate (EGCG), a catechin found in green tea, has been reported to promote RpoS degradation in *E. coli*^42^. First, we tested whether EGCG can also inhibit the RpoS stress response of *Salmonella* S1 by measuring the expression of *katE*, a gene whose the transcription is primarily regulated by RpoS and that thus acts a reporter for RpoS activity^5,43^. Since the structured environment in a biofilm and the local effects of contact-dependent competition can result in strongly heterogeneous responses, we studied gene expression at the single cell level by combining fluorescent promoter fusions with flow cytometry^44^. In both monoculture and mixed culture conditions, 50 µM EGCG significantly inhibited the RpoS stress response in S1, especially in the subpopulation with a high RpoS activity (Fig. 3a). Subsequently, we studied how this reduction in RpoS activity influenced the antimicrobial tolerance. Since the strong antioxidant properties of EGCG^45^ interfere with the H_2_O_2_ treatment, it did not allow to assess the influence of RpoS inhibition on H_2_O_2_ tolerance. In addition, it has also been reported that production of reactive oxygen species contributes to the antimicrobial activity of ciprofloxacin^46^. However, we did not observe an induction of reactive oxygen species upon ciprofloxacin treatment in our model system (Supplementary Fig. 2). Moreover, EGCG did not significantly impact the level of reactive oxygen species in absence or presence of ciprofloxacin. Therefore, we focused on tolerance to ciprofloxacin. Inhibition of the RpoS stress response significantly reduced the ciprofloxacin tolerance in the polymicrobial biofilm, whereas it had only minor effects in monoculture conditions. Consequently, tolerance was no longer increased in mixed culture compared to the monoculture level. We also compared the effect of 50 µM EGCG to the genomic deletion of *rpoS* and found both to behave highly similar (Fig. 3b, Supplementary Fig. 3). Moreover, EGCG did not alter the tolerance of the Δ*rpoS* deletion mutant (Supplementary Fig. 4), further supporting that EGCG suppresses the induction of antimicrobial tolerance in polymicrobial biofilms via inhibition of the general stress response. In contrast to the first strategy, absolute cell counts confirmed that pretreatment with EGCG also resulted in a lower absolute number of S1 cells surviving the ciprofloxacin treatment in mixed-culture conditions (Fig. 3c, Supplementary Fig. 3).

### Reducing biofilm density weakens competitive interactions and completely inhibits the enhanced tolerance of polymicrobial biofilms

**Fig. 4:**
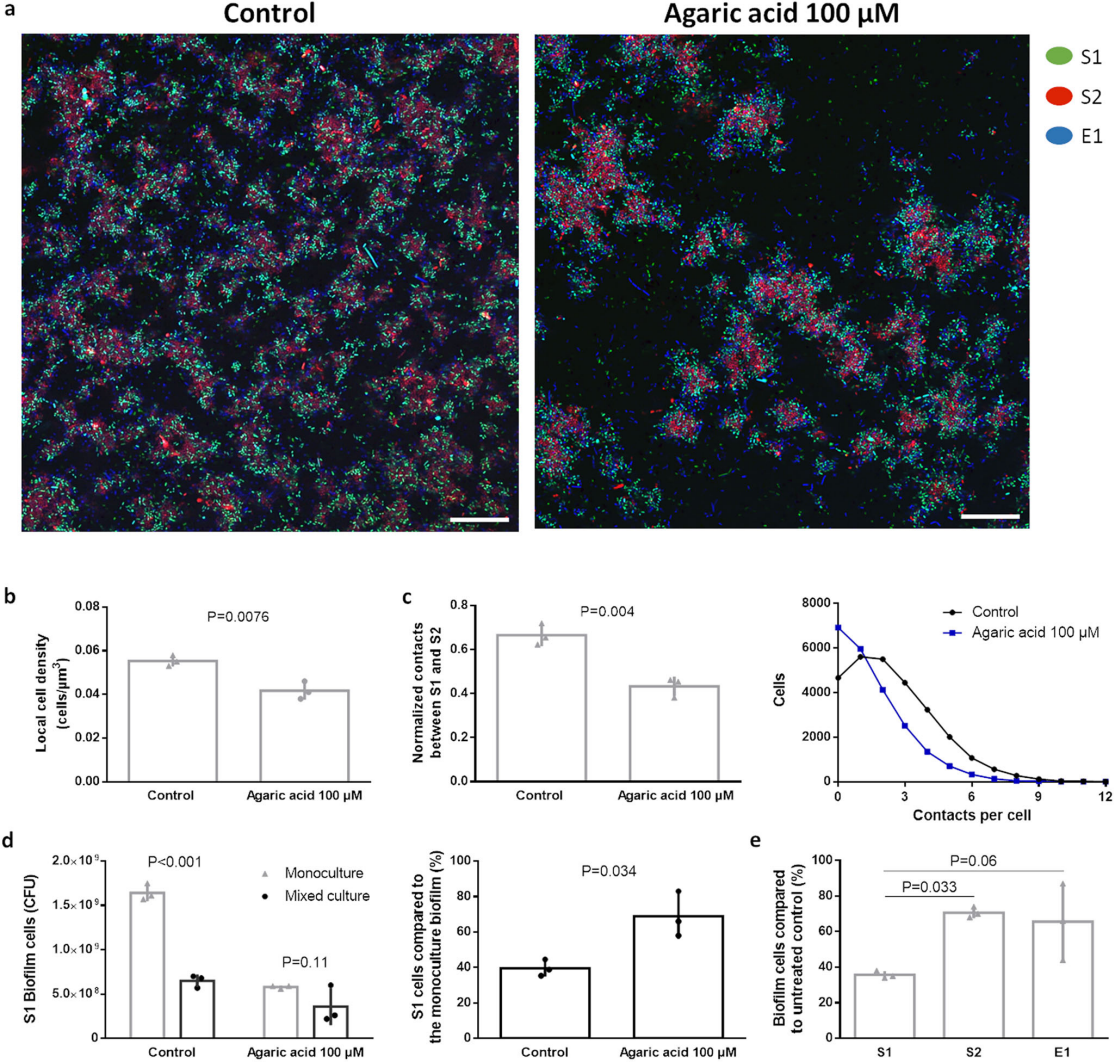
Agaric acid reduces biofilm density and weakens the competitive interactions in polymicrobial communities. **a** Agaric acid alters the biofilm structure in mixed culture conditions. S1, S2 and E1 are shown in green, red and blue respectively. Contrast was increased manually for visualization purposes. Scale bars are 25 µM. **b** Preventive treatment with 100 µM agaric acid reduces the local cell density of the polymicrobial biofilm (see Material and Methods). The mean and standard deviation of three biological repeats are shown. *P* values are calculated via a two-tailed *t* test. **c** Agaric acid reduces the contacts between S1 and S2 (see Material and Methods). The mean and standard deviation of three biological repeats are shown. *P* values are calculated via a two-tailed *t* test. In addition, the distribution of the number of contacts per cell for treated and untreated polymicrobial biofilms is displayed. **d** Agaric acid reduces the level to which S1 is inhibited by the presence of S2 and E1. The level of S1 inhibition by competition is determined by dividing the number of S1 biofilm cells in mixed culture conditions by the number of S1 biofilm cells in monoculture conditions, either in the presence of 100 µM agaric acid or a DMSO control. The mean and standard deviation of three biological repeats is shown. *P* values are calculated via a two-way ANOVA with Tukey multiple comparisons corrections or a two-tailed *t* test. **e** S1 is more sensitive to the agaric acid biofilm inhibitor than S2 and E1 in monoculture conditions. The level of biofilm inhibition by agaric acid was determined by dividing the number of biofilm cells in the presence of 100 µM agaric acid by the number biofilm cells in a DMSO control. The mean and standard deviation of three biological repeats are shown. *P* values were calculated via a one-way ANOVA with Tukey multiple comparisons corrections.

Finally, we explored a more generic strategy that does not require prior information on the attack mechanisms or competition sensing systems inducing the enhanced tolerance. It has generally been observed that competition weakens with decreasing levels of cell density because resources become less scarce and segregation between the different strains increases^47–51^. We therefore hypothesized that reducing the density of the polymicrobial biofilm can also prevent the induction of the tolerance phenotype. In further support of this hypothesis, we previously observed that the competition in our model community was significantly weaker in the more disperse planktonic phase compared to the biofilm phase. In this planktonic phase, both virulence and tolerance associated pathways were no longer induced by the presence of the other species^5^. In order to study the potential of chemically reducing the biofilm density, we utilized the biofilm inhibitor agaric acid. This compound inhibits biofilm formation without affecting growth by interfering with flagellar rotation and thus preventing surface attachment^52^. A preventive treatment with 100 µM agaric acid resulted in a sparse polymicrobial biofilm characterized by reduced local cell density (Fig. 4a, b). Since competition in our model system is mainly driven by T6SS, we also determined the number of contacts between S1 and S2 in the polymicrobial biofilm. Bacteria were considered in contact if the distance between their respective cell walls is 0.75 µm or lower, the expected average length of the T6SS^53^. Agaric acid was found to decrease the number of contacts between S1 and S2 by 30%, even if we normalized for the lower number of cells present (Fig. 4c). The reduction in biofilm density resulted in a 30% lower inhibition of S1 by the other two strains (Fig. 4d). This reduced level of competition experienced by S1 cannot be due to preferential biofilm inhibition of the other competitors, as S1 is the most sensitive to agaric acid in monoculture conditions (Fig. 4e). These results therefore indicate that agaric acid can reduce competition by lowering the density of polymicrobial biofilms.

**Fig. 5:**
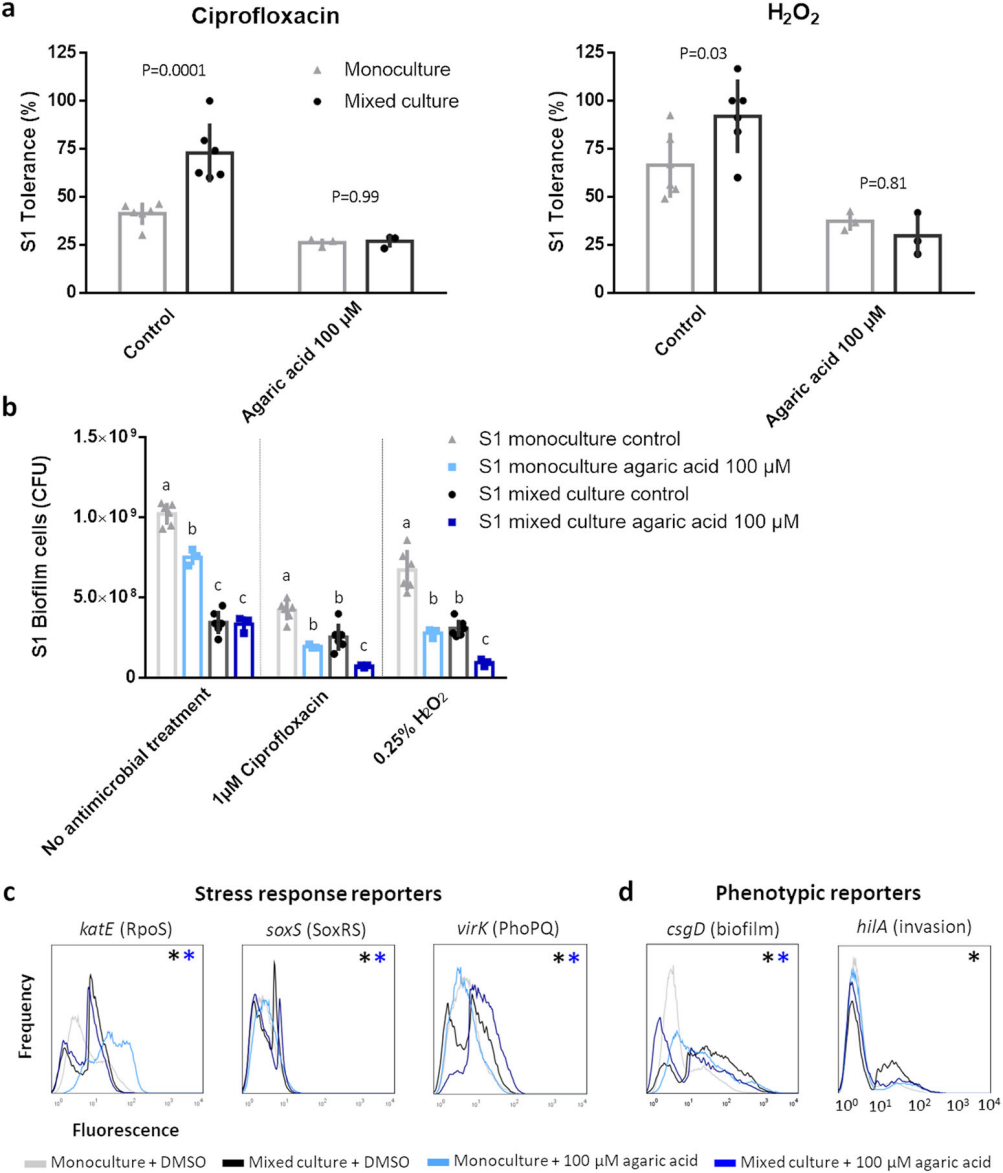
Agaric acid prevents the competitive response and abrogates the increased tolerance of S1 in polymicrobial biofilms. **a** The tolerance of S1 is no longer enhanced in the presence of S2 and E1 if the biofilm density is reduced via a preventive agaric acid treatment. S1 tolerance is calculated as the ratio between the number of antimicrobial treated and mock treated S1 biofilm cells, either in monoculture or mixed culture conditions. The mean and standard deviation of three to six biological repeats are shown. *P* values are calculated via a two-way ANOVA with Tukey multiple comparisons corrections. **b** The combination of agaric acid and a traditional antimicrobial results in the lowest absolute number of S1 cells surviving the treatment. The mean and standard deviation of three to six biological repeats are shown. Significant differences are calculated via a two-way ANOVA with Tukey multiple comparisons corrections. Different superscript letters indicate significant number of S1 cells surviving treatment, determined per antimicrobial treatment (*P* < 0.05). **c** Agaric acid inhibits the activation of the RpoS stress response. The FACS profiles show the population distribution of S1 fluorescence under the different conditions. In each condition, 100,000 cells are analyzed. Data are analyzed by using the FlowJo software. Significant differences between expression profiles are determined via probability binning as described in materials and methods. Significant differences between monoculture and mixed culture conditions with the addition of DMSO are indicated with a black asterisk whereas differences between monoculture and mixed culture conditions in the presence of agaric acid are indicated with an blue asterisk. One representative repeat of two biological repeats is shown. **d** The reduced competition in presence of agaric acid prevents the induction of *csgD* and *hilA* in mixed culture conditions. The FACS profiles show the population distribution of S1 fluorescence under the different conditions. In each condition, 100,000 cells are analyzed. Data are analyzed by using the FlowJo software. Significant differences between expression profiles are determined via probability binning as described in materials and methods. Significant differences between monoculture and mixed culture conditions with the addition of DMSO are indicated with a black asterisk whereas differences between monoculture and mixed culture conditions in the presence of agaric acid are indicated with an blue asterisk. One representative repeat of two biological repeats is shown.

We then determined how the reduction in biofilm density and competition influenced the antimicrobial tolerance in mixed culture conditions. As hypothesized, the weakening of the competitive interactions in the polymicrobial biofilm reduced the tolerance of S1 to the monoculture level (Fig. 5a, Supplementary Fig. 5). Moreover, in contrast to the competition quenching strategy that inhibited the T6SS of S2 directly, a lower absolute number of S1 cells survived the antibiotic treatment in mixed culture conditions compared to the communities without agaric acid (Fig. 5b, Supplementary Fig. 5). The decrease in tolerance thus overrode the reduction in competition, resulting in an effective combination treatment. Subsequently, we aimed to validate that a weakened induction of the stress response systems could underlie the reduced tolerance of polymicrobial biofilms treated with agaric acid (Fig. 5c). To measure the activity of these stress response systems, we monitored the expression of downstream genes that are primarily regulated by the stress response in question^5^. Unexpectedly, the reporter genes for the PhoPQ and SoxRS stress responses showed a stronger induction in the polymicrobial communities with agaric acid. However, we observed that the general stress response RpoS was no longer activated in the presence of S2 and E1. This was not due to agaric acid directly altering the physiology of *Salmonella* as agaric acid did not significantly reduce *katE* expression in stationary phase monocultures (Supplementary Fig. 6). While the lack of RpoS induction in mixed culture conditions could be partly due to agaric acid increasing the activity of RpoS in monoculture conditions, the absolute level of RpoS activity in mixed culture conditions was also lower in the presence of agaric acid compared to the mixed culture untreated control. Agaric acid thus reduced both the induction and the overall activity of the general stress response in mixed culture conditions. Although overall effects were small, this minor reduction in RpoS activity could contribute to the lack of competition-induced tolerance in the agaric acid treated biofilms.

Finally, we also explored the effect of this reduced competition on the expression of pathways regulating biofilm formation and epithelial invasion. In *Salmonella*, CsgD is the key regulator of biofilm formation^35^, while HilA controls the expression of the invasion genes encoded on *Salmonella* Pathogenicity Island 1 (SPI-1)^28^. Previously, we observed that both *csgD* and *hilA* showed an increased expression in presence of competitors and that this upregulation resulted in an enhanced biofilm matrix production and epithelial invasion in mixed culture conditions^5^. Promisingly, 100 µM agaric acid completely nullified the induction of both *csgD* and *hilA* in the polymicrobial community (Fig. 5d). The lack of *csgD* induction could also partly explain the reduced tolerance in mixed culture conditions as biofilm formation is strongly associated with high levels of tolerance^26^. In addition, these results indicate that the entire defensive response could be quenched if the level of competition is reduced. Competition quenching strategies could thus also be useful to decrease virulence traits such as biofilm formation and epithelial invasion in polymicrobial communities.

## Discussion

Recent insights into the social interactions in polymicrobial communities indicate that competition among different bacterial species can increase tolerance and virulence^5^. Bacteria have adapted their stress response systems to sense the damage caused by competition^18^. Since these same stress response systems also regulate the expression of tolerance and virulence phenotypes^21,25^, the induction of a stress response by competition could thus contribute to the worse clinical outcome associated with the polymicrobial infections^3,30,54,55^. Therefore, we here explored different types of competition quenching strategies that interfere with this cascade in order to prevent the increase in antimicrobial tolerance in response to competition (Fig. 1).

First, we showed that genetically inactivating the T6SS of surrounding competitors completely prevented the induction of antimicrobial tolerance in our focal *Salmonella* strain. Such a strategy could be widely applicable as T6SS are highly common in relevant competitive environments such as the human gut^56^ and stress response systems have been shown to protect against T6SS-mediated attacks in other pathogens such as *E. coli*, *P. aeruginosa* and *Vibrio cholera*^19,20^. Several chemical inhibitors of T6SS that could support this antimicrobial strategy have also been identified already, including antibodies^57^, a biomimetic cyclic peptide^58^ and small-molecule compounds^59^. In addition, this strategy of targeting microbial attack could be expanded to other common bacterial weapons known to induce a stress response such as antibiotics^21^ and bacteriocins^22^. However, as highlighted in this work, such approach can also have significant disadvantages. We found that the decrease in competitive inhibition by surrounding bacteria is too large to be compensated by the reduction in tolerance. Consequently, a similar number of pathogens survived the antimicrobial treatment, resulting in an ineffective combination treatment. In addition, directly inhibiting bacterial weapons is likely not an optimal antimicrobial strategy in complex bacterial communities with various competing species. In these diverse communities, it is probable that several attack mechanisms will trigger defensive responses, thus requiring multiple compounds to target the different individual attack mechanisms.

Second, we evaluated a competition quenching strategy that circumvents both pitfalls by directly inhibiting the stress response systems that can sense competition. We previously showed that *Salmonella* activates the general (RpoS) and the oxidative (SoxRS) stress responses in response to competition and that genomic deletion of either RpoS or SoxRS prevented the induction of the downstream response to competition^5^. Here we opted to target the general stress response because a chemical inhibitor of RpoS has previously been identified^42^. RpoS inhibition completely abolished the enhanced tolerance in mixed culture conditions. These results further support that interfering with only one of the competition sensing stress responses is sufficient to completely inhibit the increased tolerance of *Salmonella* in response to competition. This prudent co-regulation requiring activation of both stress responses limits the conditions under which a tolerance phenotype is induced by competition. Separately, the RpoS and SoxRS stress responses could be activated in a wide variety of environments, including conditions where competitive interactions with other bacterial strains are not a dominant factor. For example, RpoS is also strongly upregulated in monoculture populations reaching stationary phase^60^, whereas the reactive oxygen species that activate SoxRS can also be generated by abiotic stressors such as heat and metals^31^. The tight regulatory control of the competitive response by several stress responses could be due to the high cost associated with the inopportune expression of defensive responses such as biofilm formation and invasion^61,62^. Simultaneously, this prudent regulatory system thus also allows to only interfere with one of the competition sensing stress responses in order to prevent the induction of the downstream response, increasing the feasibility and potential of this competition quenching strategy. In addition, although only a limited effect on ciprofloxacin tolerance in monoculture biofilms was observed in our set-up, directly targeting stress responses would likely reduce tolerance in the absence of competitors for other combinations of pathogens and antimicrobials since several stress responses have also been associated with enhanced antibiotic tolerance in monoculture conditions^21,24^. However, in contrast to traditional antimicrobial treatments, the effect of stress response inhibition strategies is expected to be enhanced in polymicrobial communities rather than reduced. Moreover, competition sensing inhibition could also increase the efficacy of antimicrobial strategies that utilize beneficial bacteria, such as probiotics, to compete with pathogens since the pathogen would not be able to adapt its behavior to the presence of these competitors. While competition sensing is expected to have broadly evolved^18^ and stress response systems are partly conserved among different species, they also have organism-specific functions, particularly for virulence-associated traits^63^. It thus remains to be elucidated to what extent this competition sensing inhibition strategy needs to be tailored towards the specific pathogen in question. Moreover, which stress responses are activated in a polymicrobial community will depend on the competition-associated stressors present. It is thus possible that different stress response systems need to be targeted depending on the community composition. However, it is also expected that some stressors such as nutrient depletion and oxidative stress are highly prevalent in a wide variety of communities^18^.

Finally, we also tested an approach that does not require any prior knowledge about the attack or competition sensing mechanisms involved in the competitive response, but rather reduces the overall level of competition in the polymicrobial biofilm. Competition decreases with (i) reduced levels of cell density, (ii) increased resource availability, and (iii) stronger spatial segregation between the different strains^5,47,48^. We opted to focus on reducing the biofilm density as this approach has the additional benefit that it can decrease antimicrobial tolerance in monoculture conditions^52,64^ and various biofilm inhibition strategies are already available^65,66^. Similar to the two previous strategies, no increased tolerance compared to monoculture conditions was observed if the biofilm density was reduced. Moreover, reducing the level of competition also reduced the expression of pathways that regulate other virulence-associated traits such as biofilm formation and epithelial invasion. It is possible that reducing density mainly inhibits competition in communities where contact-dependent competition prevails. However, such strategies would still be widely applicable as contact-dependent mechanisms are highly common in relevant competitive environments such as the human gut^56^. In addition, the distance between competitors is also an important factor for other competitive mechanisms such as the secretion of toxins^60,67^, although potentially to a lesser extent than in the case of contact-dependent inhibition.

These competition quenching strategies can serve as a counterpart to the well-studied quorum quenching therapies^33,34^ as competition quenching targets inter-strain competition instead of intra-strain cooperation. The most favorable strategy depends on the relative effect of competition and cooperation on the pathogenicity and tolerance of a given pathogen in its natural environment. Inhibiting the attack mechanism of surrounding competitors is analogous to the signal supply inhibition of quorum quenching therapies^29,33^. Quorum sensing systems can vary in specificity, responding either to one specific signal or to multiple signals^68^. Targeting the signals of these promiscuous detection systems poses similar challenges as those faced by competition quenching strategies that inhibit attack mechanisms since in diverse communities surrounding bacteria can still activate these detection systems. However, targeting the signals of detectors with high specificity can circumvent this disadvantage^68^. Conversely, directly inhibiting the competition sensing systems aligns with quorum quenching therapies that target the quorum sensing detectors. Similarly to the response to competition being regulated by several stress responses^5^, bacteria typically have access to multiple signaling networks. Some of these systems work redundantly, whereas others require simultaneous activation of the different signaling networks^68^. Similar to competition sensing inhibition strategies, targeting these latter systems will also require only one inhibitor to shut down the complete response. Competition quenching and quorum quenching strategies can also be tightly linked. For example, reducing cell density might not only reduce competition but can also decrease the induction of virulent phenotypes by quorum sensing^69^. In addition, quorum sensing often regulates toxin production as these toxins are more effective at high densities^70^. Quorum quenching therapies could thus also reduce the overall level of competition in a community, especially if these quorum sensing-regulated toxins would induce a tit-for-tat response in surrounding bacteria^23,71^.

One of the most promising aspects of quorum quenching therapies is the prediction that they can select against resistance as quorum sensing signals are both public goods themselves and often regulate the production of other public goods^34^. Since these public goods provide a shared benefit to other cells in the population, targeting public goods is expected to be evolutionarily robust. Resistant mutants do not selfishly benefit from their resistance but instead share the advantages provided by the public good with the surrounding sensitive cells^14,34^. However, in some cases resistance against quorum quenching therapies has been identified due to quorum sensing pleiotropically regulating private traits in addition to the public traits^72^. It is expected that competition sensing inhibition therapies have a similar selection pressure for resistance as quorum quenching therapies as these stress response systems most likely also regulate both private and public traits. For example, in our model community, competition sensing regulates both public traits such as biofilm formation^62^ and epithelial invasion^73^ and private traits like antibiotic efflux.

We have, however, just began to unravel the role of competitive interactions and competition sensing in the increased tolerance of polymicrobial communities. For example, in contrast to the competition sensing hypothesis, it was shown that cell damage and resource limitation were not strong cues for antibiotic induction in *Streptomyces*^74^. These results indicate that bacteria have developed different regulatory systems to integrate cues from their social environment and adapt their behavior. More knowledge on the prevalence of competition sensing and competition-induced tolerance in different species and under various conditions is thus required to estimate the potential and viability of strategies directed to quench competition. In addition, we here provided an initial proof of concept in a well-characterized yet relatively simple in vitro community. Competition quenching strategies will need to be validated in more diverse communities that include multiple competitors with different attack mechanism and in more complex (in vivo) conditions. However, in an era where traditional antibiotics no longer suffice due to the rapid rise of resistance, our exploratory study indicates that competition quenching treatments provide an appealing direction worth to further investigate.

## Methods

### Bacterial strains and growing conditions

The bacterial strains and plasmids used in this study are listed in Tables 1 and 2. Overnight cultures of *S*. Typhimurium SL1344 (S1), *S*. Typhimurium ATCC14028 (S2), or *E*. *coli* MG1655 (E1) strains were grown in lysogeny broth (LB) at 37 °C with 100 μg/ml ampicillin and continuously shaking at 200 rpm.

**Table 1:** Overview of the strains utilized in this study

Strain	Source
*Salmonella* Typhimurium SL1344 (S1)	^82^
*Salmonella* Typhimurium SL1344 Δ*rpoS* (S1 Δ*rpoS*)	^83^
*Salmonella* Typhimurium ATCC14028 (S2)	ATCC
*Salmonella* Typhimurium ATCC14028 Δ*clpV* (S2 ΔT6SS)	^5^
*Escherichia coli* MG1655 (E1)	ATCC

**Table 2:** Overview of the plasmids utilized in this study

Plasmids	Source	Description
pFPV25	^82^	Promoter-trap vector constructed by inserting an EcoRI-HindIII fragment containing a promoterless *GFPmut3* into plasmid pED350 (colE1, *bla*, *mob*); Ap^R^
pFPV25.1_GREEN	^82^	0.6 kb *Sau3AI* fragment inserted in the BamHI site of pFPV25, containing the promoter region of S. Typhimurium *rpsM* encoding for the ribosomal protein S13 (constitutive promoter); Ap^R^
pFPV25.1_RED	^5^	Identical to pFPV25.1_GREEN; *GFPmut3* gene replaced by *dsRed*.T4 gene
pFPV25.1_BLUE	^5^	Identical to pFPV25.1_GREEN; *GFPmut3* gene replaced by *mtagBFP* gene
pCMPG5521	^84^	pFPV25 plasmid with the promoter region of *csgD* inserted in front of the *GFPmut3* gene
pCMPG5426	^85^	pFPV25 plasmid with the promoter region of *hilA* inserted in front of the *GFPmut3* gene
pCMPG10021	^75^	pFPV25 plasmid with the promoter region of *katE* inserted in front of the *GFPmut3* gene
pCMPG11406	^5^	pFPV25 plasmid with the promoter region of *virK* inserted in front of the *GFPmut3* gene
pCMPG11407	^5^	pFPV25 plasmid with the promoter region of *soxS* inserted in front of the *GFPmut3* gene

### Biofilm competition experiments

The overnight cultures of S1, S2, and E1 were normalized to an OD_595_ of 3.2 (±1.2 × 10^10^ cells/ml). The normalized overnight cultures were diluted 1:100 in small petri dishes (60 mm diameter, Greiner Bio-One) containing 10 ml of diluted tryptic soy broth (1.5 g/l, BD Biosciences) with ampicillin (Ap, 100 µg/ml). Monoculture or mixed culture biofilms were grown statically for 48 h at 25 °C on the bottom of the petri dishes. The same total number of cells was inoculated in monoculture and mixed culture conditions. If appropriate, 100 µM agaric acid (Sigma, dissolved in DMSO) or 50 µM Epigallocatechin gallate (EGCG) (TCI, dissolved in DMSO) was added from the beginning. In order to take into account potential effects from the DMSO solvent, a corresponding amount of DMSO (0.1% v/v) was added to the control samples. After incubation, the liquid above the biofilms was poured off and 1 ml of phosphate-buffered saline was added (PBS; 1.24 g/l K_2_HPO_4_, 0.39 g/l KH_2_PO_4_, 8.8 g/l NaCl). Afterwards, the biofilm cells were scraped off the bottom of the dish with a cell scraper (Greiner). In order to disrupt remaining clumps and obtain single cells, the samples were vortexed and passed through a syringe (25 G). The number of colony forming units (CFU) of each strain was determined by plating. To differentiate between the strains, S1 was labeled with constitutive GFPmut3 on a plasmid, while S2 and E1 were labeled with a plasmid-encoded constitutive dsRed.T4 protein. Differences in colony shape and size allowed to differentiate between S2 and E1 during CFU counting.

### Tolerance assays

Monoculture and mixed culture biofilms were grown on the bottom of petri dishes for 48 h at 25 °C as described above. The medium was then replaced by 5 ml PBS (mock treatment), 0.25% H_2_O_2_ or 0.33 µg/ml ciprofloxacin and the biofilms were incubated for an additional hour at 25 °C. Subsequently, the biofilms were resuspended in 1 ml PBS, scraped off and plated out on LB agar plates for CFU determination as described above. Tolerance was calculated as the ratio of the S1 biofilm cells after the antimicrobial treatment and the mock treatment.

### Biofilm gene expression

Gene expression was measured by combining fluorescent promoter fusions encoded on a plasmid and flow cytometry as described previously^5,75^. Hereto, monoculture or mixed culture biofilms were inoculated with the different S1 reporter strains. After 48 h of incubation, biofilm cells were collected as described above and the fluorescence of 100,000 cells was measured at single cell level using the BD Influx (Becton Dickinson). Prior to each analysis a calibration was performed using SPHEROTM Rainbow Calibration Particles, 8 peaks, 3.0–3.4 µm (Spherotech), according to the manufacturer’s recommendations. The difference in expression profile between various conditions was compared via probability binning. Probability binning divides the FACS profile of the control sample in bins with an equal number of events. Subsequently, these bins are also applied to the test sample. A Cox Chi-square test compares the number of events between test and control sample in each corresponding bin. The Chi Squared value is next conversed to a normalized T(χ) metric that is analogs to a t-score and describes the similarity between two distributions^76^. Biologically relevant T(χ) thresholds for each fluorescent promoter fusion were determined previously based on the variance of the reporter gene in question^5^.

### Planktonic gene expression

Overnight cultures of S1 pCMPG10021 were normalized to an OD_595_ of 3.2 (±1.2 × 10^10^ cells/ml) and diluted 1:100 in 5 ml of diluted tryptic soy broth (1.5 g/l, BD Biosciences) with ampicillin (Ap, 100 µg/ml). The cultures were grown for 24 h at 25 °C and 200 rpm. The expression of GFP under the control of the *katE* promoter was measured using the CytoFELX flow cytometer (Beckman Coulter). Capture and the initial analysis of the data was performed in CytExpert v2.5.

### Oxidative stress measurements

To measure the oxidative stress induced during antibiotic treatment, we used the ROS-Glo H_2_O_2_ Assay (Promega). Briefly, overnight cultures of S1 and S1ΔrpoS, were normalized to an OD_595_ of 3.2 (±1.2 × 1010 cells/ml). The normalized overnight cultures were diluted 1:100 in 200 µl of diluted tryptic soy broth (1.5 g/l, BD Biosciences) containing ampicillin (Ap, 100 µg/ml). In order to determine the effect of EGCG on oxidative stress during antibiotic treatment, 50 µM Epigallocatechin gallate (EGCG) (TCI, dissolved in DMSO) was added, whereas a corresponding amount of DMSO (0.1% v/v) was added to the control samples. The biofilms were incubated statistically at 25 °C in a flat-bottom 96-well plate (Greiner Bio-One International). After 48 h, the medium above the biofilm was replaced with 100 µl PBS (mock treatment) or 0.33 µg/ml ciprofloxacin. Either treatment also contained 20 µl of ROS-Glo™ H_2_O_2_ Substrate solution. The biofilms were incubated for an additional hour at 25 °C. The rest of the assay proceed according to the manufacturer’s instructions (employing the non-lytic assay) and the luminescence was measured using the Synergy Mx Microplate Reader (BioTek).

### Image acquisition

For microscopic analysis, mixed culture biofilms were grown in µ-Slide 8 Wells (Ibidi). Hereto, differentially labeled fluorescent strains were incubated for 48 h at 25 °C in 300 µl diluted TSB containing 100 μg/ml of ampicillin and 100 µM agaric acid or the corresponding amount of DMSO. Image stacks were acquired with a Zeiss LSM 880 confocal microscope combined with an Airyscan detector using a 40x water objective (LD C-Apochromat 40x/1.1 W Korr M27, Zeiss) for visualization and a 63x oil objective (Plan-Apochromat 63x/1.4 Oil DIC M27, Zeiss) for further processing. The physical resolution of the 40x images was set to 90 nm in the xy-plane and for the 60x images to 70 nm in the xy-plane and 143 nm in the z-direction. Images were obtained in the FastAiryscan mode respectively using a 405 nm, 488 nm and 561 nm laser for visualization of *mtagBFP* (E1), *GFPmut3* (S1) and *dsRed*.T4 (S2).

Image processing Image stacks were initially split in a set of smaller image substacks using a 7-by-7 grid in the xy-plane with a 20% overlap in both directions. Every substack was filtered using a hessian-based Frangi vesselness filter enhancing blob-like structures. Subsequently, image substacks were binarized using a threshold scaled with 0.07, the threshold obtained from Otsu’s method^77^. Binarized image substacks were stitched together and subsequently segmented and labeled using 3D imaging suite^78^ implemented in the ImageJ platform^79^. Labeled objects were parameterized by three radii based on the eigenvalues of the 3D moment matrix^78^. From the three radii, the bacteria were characterized as spherocylinders keeping the volume constant. Potential contacts between S1 and S2 were acquired by multigrid contact detection^80^ and further resolved using Mpacts software^81^. Contacts were retained if the distance between spherocylinders did not exceed 0.75 μm, the expected length of a T6SS^53^. The number of contacts was normalized by the total sum of S1 and S2 cells. The local cell density was obtained for every cell by counting the number of cells in the surrounding spherical neighborhood with a radius of 5 μm and dividing by the volume of the neighborhood.

### Reporting summary

Further information on research design is available in the Nature Research Reporting Summary linked to this article.

### Supplementary information

Supplementary Information

Reporting Summary

## Data Availability

Work presented in this paper used Mpacts software. To ensure reproducibility, we make use of the Docker (https://www.docker.com/) platform. Our reproducibility package (https://gitlab.kuleuven.be/mebios-particulate/competition_quenching_2022) can be automatically recreated executing a bash script (reproduce.sh). Raw microscopy images are available upon reasonable request. Further information and requests for resources should be directed to and will be fulfilled by the corresponding author, Hans Steenackers (hans.steenackers@kuleuven.be).
