# Supplementary material for: Competition quenching strategies reduce antibiotic tolerance in polymicrobial biofilms
Source: NPJ Biofilms Microbiomes. 2024 Mar 19;10:23. doi: 10.1038/s41522-024-00489-6 (PMC10951329; doi:10.1038/s41522-024-00489-6)
Supplement: Supplementary file 1 — Supplementary Information [file 41522_2024_489_MOESM1_ESM.pdf]

Supplementary information

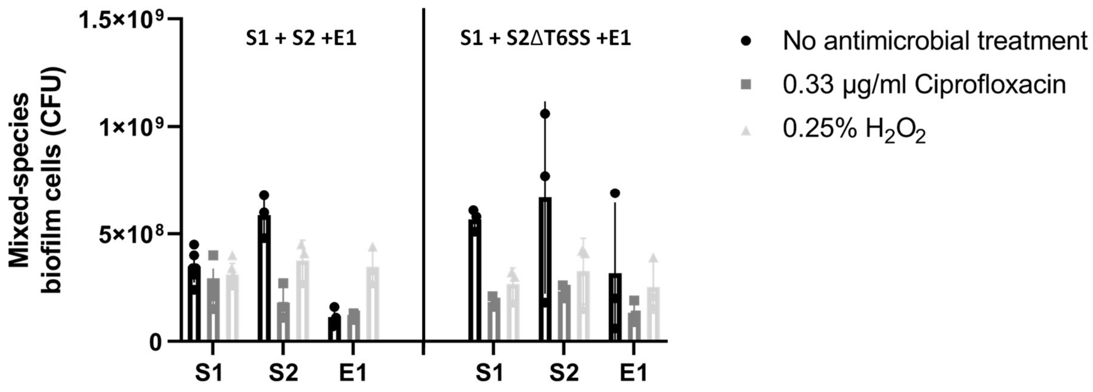

Supplementary Figure 1: The community response to antimicrobial treatment in the wildtype community and the community containing a T6SS-deficient S2 mutant. The mean and standard deviation of three biological repeats are shown.

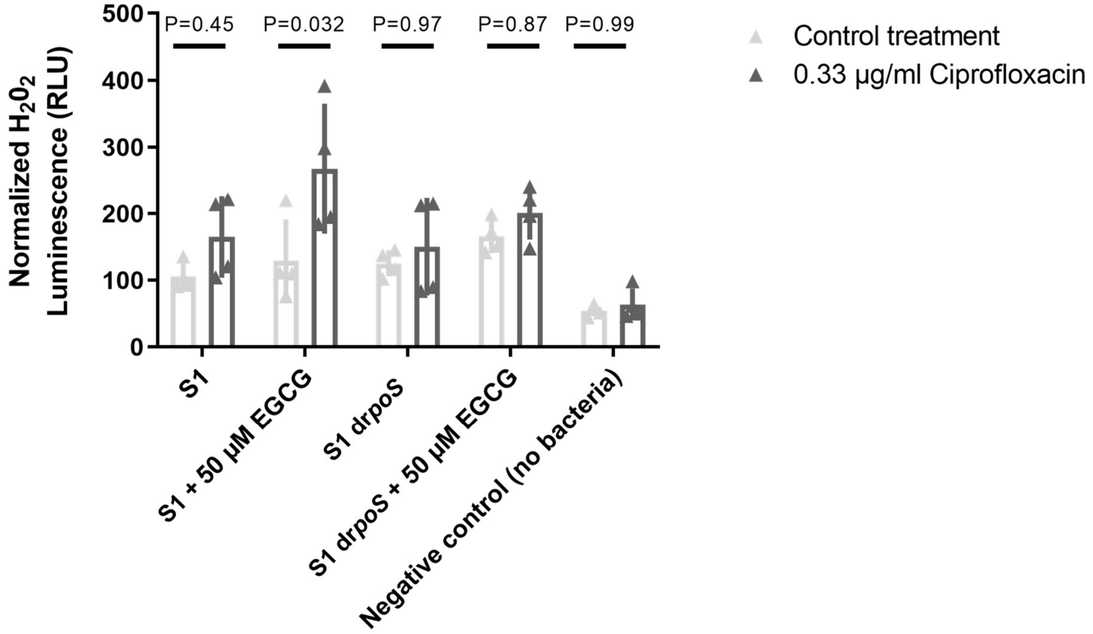

Supplementary Figure 2: EGCG does not interfere with the ciprofloxacin treatment as hydrogen peroxide production is not induced during treatment. The mean and standard deviation of three biological repeats are shown. Significant differences are calculated via a one-way ANOVA with Tukey multiple comparisons corrections.

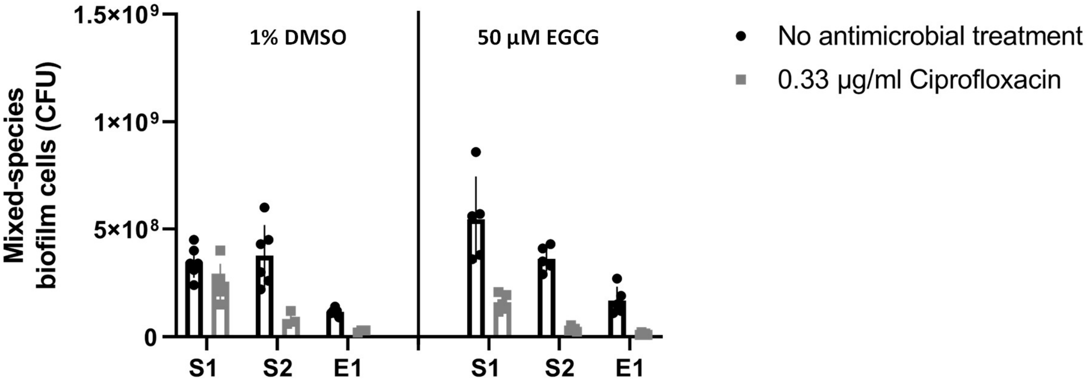

Supplementary Figure 3: The Community response to antimicrobial treatment in the presence and absence of 50 µM EGCG. The mean and standard deviation of three biological repeats are shown.

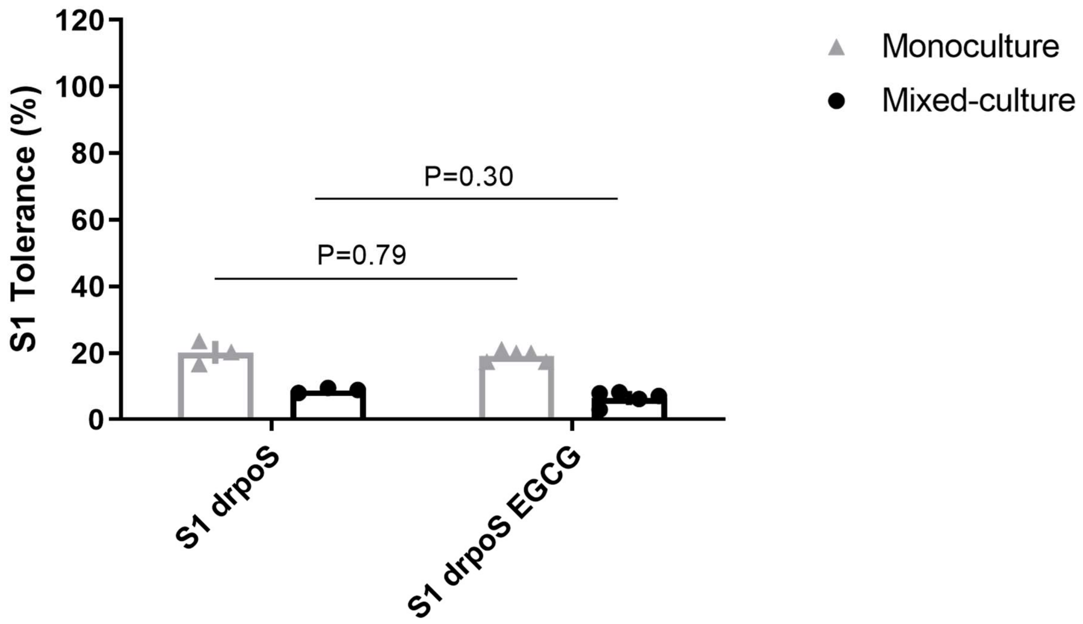

Supplementary Figure 4: EGCG has no effect on the ciprofloxacin tolerance of a S1 Δ*rpoS* deletion mutant. Tolerance is calculated as the ratio between the number of antimicrobial treated and mock treated S1 biofilm cells, either in monoculture or mixed culture conditions. The mean and standard deviation of three to six biological repeats are shown. P-values were calculated via a one-way ANOVA with Tukey multiple comparisons corrections.

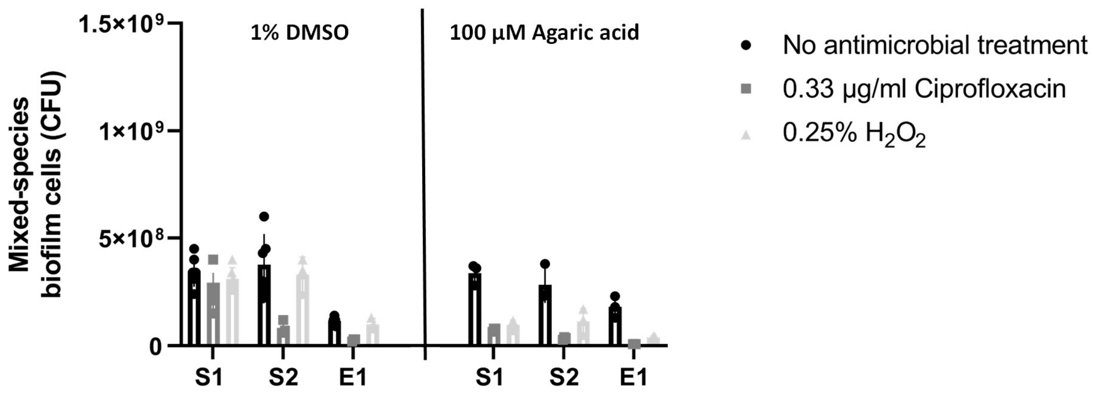

Supplementary Figure 5: The community response to antimicrobial treatment in the presence and absence of 100 µM agaric acid. The mean and standard deviation of three biological repeats are shown.

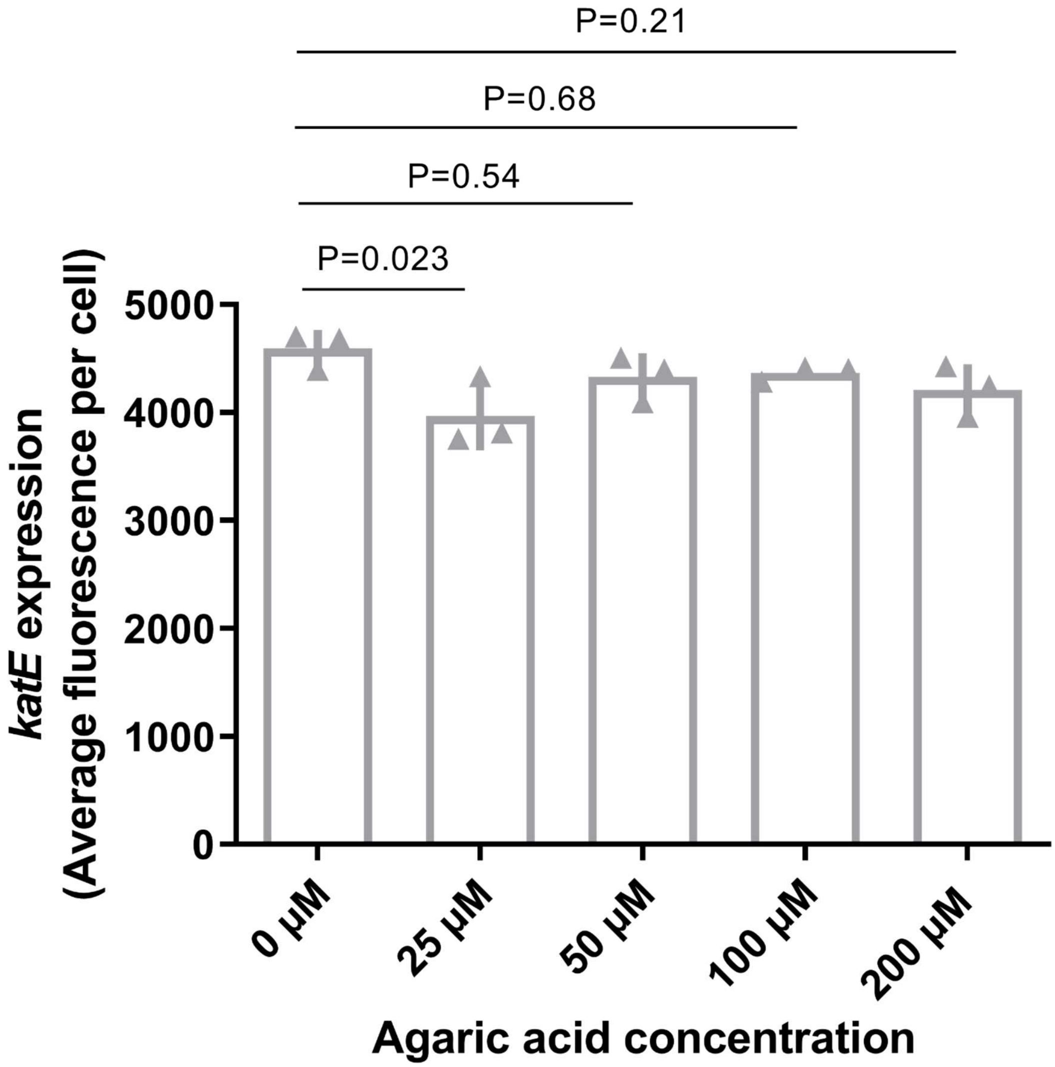

Supplementary Figure 6: Agaric acid does not directly impact RpoS activity in planktonic S1 monocultures. Fluorescence of the *katE* reporter fusion was measured for 30 000 cells per sample using flow cytometry. The mean and standard deviation of three biological repeats are shown. P-values were calculated via a one-way ANOVA with Tukey multiple comparisons corrections
